# Mind the guideline gap: emergent CT in patients with epilepsy for trauma rule-out—A retrospective cohort study

**DOI:** 10.1186/s42466-025-00370-7

**Published:** 2025-02-24

**Authors:** Kristina Szabo, Udo Obertacke, Vesile Sandikci, Sarah Ghanayem, Angelika Alonso, Johann S. Rink, Annika Marzina, Michael Platten, Carolin Hoyer

**Affiliations:** 1 Department of Neurology, Medical Faculty Mannheim, University Medical Centre Mannheim, Heidelberg University, Theodor-Kutzer-Ufer 1-3, 68135 Mannheim, Germany; 2 Department of Orthopaedics and Trauma Surgery, Medical Faculty Mannheim, University Medical Centre Mannheim, Heidelberg University, Mannheim, Germany; 3 Department of Radiology and Nuclear Medicine, University Medical Centre Mannheim, Heidelberg University, Mannheim, Germany

**Keywords:** Seizure, CT, Trauma, Emergency neurology

## Abstract

**Background:**

Patients with epileptic seizures represent a significant proportion of emergency department (ED) admissions and are often referred for cranial imaging due to suspected or observed trauma. Neurological guidelines provide limited advice on indications for imaging in this scenario, and traumatological clinical decision rules on the use of CT in mild traumatic brain injury explicitly exclude patients with seizures preceding the trauma. This gap in recommendations may contribute to overimaging for trauma rule-out after a seizure.

**Methods:**

We analysed medical records of patients with known epilepsy admitted to our ED after a seizure between January 2022 and March 2024. Using clinical data including the findings from cranial CT and risk factors for traumatic brain injury, we re-assessed the need for CT imaging by application of the Canadian CT head rule (CCHR) or in the context of head trauma under anticoagulation.

**Results:**

During the observational period, 683 patients with known epilepsy were referred to our hospital due to a seizure (mean age 48.8 years, 57.7% male). A head CT scan was obtained in 337 (49.3%) of all encounters. In only two patients, CT diagnosed an acute seizure-related traumatic lesion, one focal subarachnoid haemorrhage and one skull base fracture. Twenty-six cases (3.8%) with seizure-related trauma were reassessed as requiring a CT for trauma-related injury evaluation. Particularly in the absence of head impact or risk factors, a high degree of variability regarding CT ordering practice was observed.

**Conclusions:**

Our results demonstrate frequent use and low diagnostic yield of CT in ED seizure patients with respect to trauma-related head injury. Circumstantial factors, clinical signs or symptoms and medical risk factors variedly impact on clinicians’ decision to perform imaging. The absence of clear recommendations regarding imaging for trauma apparently provokes frequent diagnostic rule-out even in patients with low risk for traumatic brain injury. We suggest an approach to identify patients not requiring a head CT by considering the CCHR, presence of anticoagulation and appreciating the postictal state as a feature specific to patients with seizures.

## Background

Non-contrast head computed tomography (CT) has become one of the most frequently ordered diagnostic investigations in the emergency department (ED), and numbers are still on the rise [3, 20]. Its perceived usefulness and wide availability have led to a high degree of clinicians` reliance on the procedure, frequently resulting in overuse. The excessive utilization of head CT in the emergency evaluation of patients with dizziness or headache are pertinent examples in this regard [13, 16, 29]. Their scrutinous recognition has furthered a refinement of criteria for rational use of imaging in these patients and the development of alternative diagnostic algorithms and guidelines [8, 9]. These efforts are aimed at reducing known individual and systemic risks and adverse consequences of CT overimaging, such as radiation exposure or the requirement for costly and psychologically burdensome follow-up investigations in the case of incidental findings [7].

Patients with epileptic seizures comprise a substantial proportion of presentations to neurology in emergency departments [14, 35]. Indications for emergent imaging in this population are the identification of a structural aetiology or of an acute pathology in need of urgent treatment. The latter includes intracranial injury due to actual or suspected seizure-associated trauma. In this regard, the guideline of the German Neurological Association explicitly, albeit briefly, discusses the diagnostic value of an emergent head CT examination and recommends it be performed in patients in the wake of a seizure-related fall and concomitant prolonged impairment of consciousness, a new focal neurological deficit or risk factors for intracranial trauma, which, however, remain unspecified [11]. At the same time, the guideline stresses judicious CT usage in light of radiation exposure risks. Further complicating the matter, the postictal state as well as the effects of sedatives may temporarily mimic clinical signs and symptoms of traumatic brain injury. Therefore, studies of clinical decision rules regarding the use of CT after mild traumatic brain injury (TBI) have specifically excluded patients if they had a primary seizure [23, 33]. Considering this lack of clear recommendations, it does not come as a surprise that the fear of overlooking seizure-related traumatic brain injury is a relevant motivator for obtaining a CT scan, contributing to overimaging in this patient population [35].

The current study intends to analyse factors leading to CT imaging in patients after a seizure as well as diagnostic outcomes with respect to traumatic injuries. Our goal is to identify determinants for CT ordering in this regard and to quantify its diagnostic yield, which in turn may improve resource utilization in the context of adequate risk–benefit assessment and tailored emergency care for this large, yet underexplored group of ED patients.

## Methods

We retrospectively analysed records patients consecutively admitted to the ED of the University Medical Centre, Mannheim, Germany, between January 1st 2022 and March 31st 2024 for neurological consultation due to an epileptic seizure. Cases were identified from ED records according to the German diagnosis-related group system (G40.x for epilepsy and recurrent seizures, G41 for status epilepticus, R56 for unspecified convulsions). Due to different imaging indications, patients with a first-ever seizure were excluded from the analysis [5]. Cases in which the seizure did not occur directly prior to ED presentation were not considered.

Electronic medical records were reviewed, and variables were collected using a standardized data form for demographic information, information concerning the seizure from prehospital medical staff or bystanders, physical and neurological examination findings and neuroimaging results. Neuroimaging findings were categorized as acute with intraparenchymal trauma-related pathology or skull fracture, or nonacute parenchymal pathology not associated with trauma, irrespective of aetiological significance. Finally, diagnoses of other trauma-related head injury were recorded.

We retrospectively identified the reason for head imaging related to TBI from medical records with respect to (a) seizure circumstances, (b) clinical signs and symptoms possibly predictive of TBI, and (c) relevant pre-existing medical conditions. These items were comprised from various clinical decision tools designed to improve head CT utilisation in adults with minor head injury [1]. We reappraised CT indications with respect to the recommendations of the American College of Emergency Physicians—their Clinical Policy published in 2023 states that the decision to perform CT in patients with minor head injury should be made in accordance with the Canadian CT Head Rule (CCHR); a CT should also be obtained in patients with minor head injury taking an anticoagulant medication [1].

The study was approved by the Ethics Committee II, Medical Faculty Mannheim, Heidelberg University, reference number 2024-838. Written informed consent was waived due to the retrospective character of the investigation.

Statistical analysis was performed using IBM SPSS Statistics Version 29. Distributions of continuous variables between groups were compared with Student’s t-test for independent samples, and distributions of categorial variables were compared using chi^2^-test or Fisher’s exact test, depending on group sizes. Statistical significance is indicated by *p* values of < 0.05. No *p* value was calculated for group sizes below five due to limited statistical power.

## Results

Data of 683 patients with the final diagnosis of an epileptic seizure consecutively admitted to our ED between January 2022 and March 2024 were included in the study. From a total of 1347 cases extracted from the ED diagnosis database, the following 681 were excluded: first-ever seizure (n = 367), uncertain diagnosis/other chief complaint (n = 197), uncompleted emergency department care/discharge against medical advice (n = 34), primary MR imaging (n = 4), and incomplete documentation (n = 62).

The mean age of the patients in our cohort was 48.8 (± 19.5) years, and 394 of all patients (57.7%) were male. A head CT scan was obtained in 337 (49.3%) of all encounters.

### Head CT findings

In the overwhelming majority of cases receiving imaging (99.4%), head CT scan did not show a seizure-related acute lesion defined as intraparenchymal trauma-related pathology or skull fracture. In one case each, a small focal (and asymptomatic) subarachnoid haemorrhage and a skull base fracture were diagnosed. Both patients were admitted for observation without further intervention and were discharged after five and six days, respectively. Nonacute parenchymal pathologies not associated with trauma and irrespective of aetiological significance for the index seizure were reported in 148 of all examinations, 89 of these were chronic vascular (ischemic or haemorrhagic) lesions.

### Indication for head CT

A comparative analysis of seizure circumstances, signs suggestive of TBI and relevant medical conditions as reasons to order a head CT is presented in Table 1. Any clinical sign or symptom predictive of possible TBI was documented 81 times (11.9%). Sixteen patients (2.3%) with prolonged somnolence either recovered within a short period of time or were diagnosed as status epilepticus by EEG. Similarly, all observed neurological deficits in our sample (n = 35; 5.1%) resolved quickly and were evaluated as Todd’s paralysis.

**Table 1:** Seizure circumstances and signs suggestive of TBI as indications for head CT and risk factors for intracerebral trauma in minor head injury, in patients with and without CT

	CT n = 337	No CT n = 346	*p*-value^a^
Male	199 (59.1%)	195 (56.4)	0.476
Age, mean (standard deviation)	51.82 (18.44)	45.80 (20.04)	** < 0.001**
*Seizure circumstances*			
Incomplete history regarding trauma	49 (14.5%)	49 (14.2%)	0.888
Head impact from history or exam	123 (36.5%)	13 (3.8%)	** < 0.001**
Other^b^	60 (17.8%)	3 (0.9%)	
*Signs and symptoms predictive of TBI*			
Repeated vomiting	1 (0.3%)	0 (0.0%)	
Any sign of basal skull fracture	9 (2.7%)	1 (0.3%)	
Prolonged somnolence	12 (3.6%)	4 (1.2%)	
Headache	17 (5.0%)	2 (0.6%)	
Postictal neurological deficit	29 (8.6%)	6 (1.7%)	** < 0.001**
*Pre-existing medical conditions*			
Age ≥ 65 years	92 (27.3%)	62 (17.9%)	**0.003**
Anticoagulation	30 (8.9%)	22 (6.4%)	0.210
Intracerebral shunt	15 (4.5%)	7 (2.0%)	0.072
Motor vehicle accident	3 (0.9%)	3 (0.9%)	
History of intracranial bleeding	42 (12.5%)	27 (7.8%)	**0.043**
Active cancer	9 (2.7%)	22 (6.4%)	**0.021**
Cognitive impairment	31 (9.2%)	60 (17.3%)	**0.002**
Alcohol/substance disorder	61 (18.1%)	36 (10.4%)	**0.004**
*Relevance of CT regarding trauma*			
CT indicated by CCHR or anticoagulation	25 (7.4%)	1 (0.3%)	

^*a*^* p-values are provided when group size is 5 or more. Statistical significance is indicated by bold print*

^*b*^* Long seizure-free interval, language barrier*

*CCHR: Canadian CT head rule, TBI: traumatic brain injury*

Although none of the investigated criteria could undoubtedly predict the referral for cerebral imaging, CT scans were ordered significantly more frequently in patients with head impact from history or exam or a postictal focal deficit. Furthermore, CT was ordered more frequently in patients 65 years and older as well as those with a history of intracranial bleeding, active cancer, cognitive impairment and substance use disorder (Table 1).

**Fig. 1:**
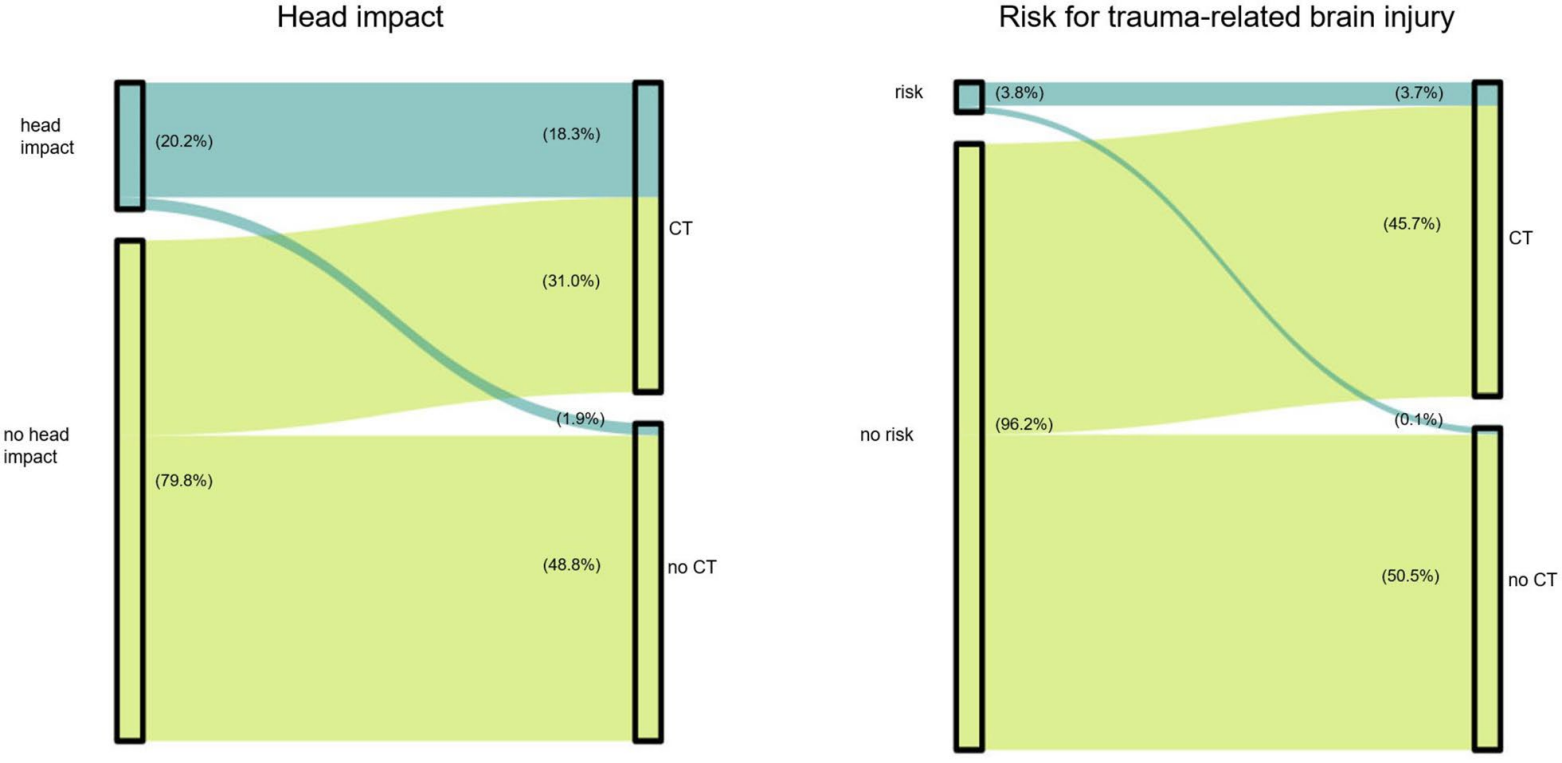
Decision to CT in the context of head impact and risk constellations. The Sankey diagrams illustrate the decision for CT in patients with or without a seizure-related head impact (left), and in patients with seizure-related trauma and, either at least one clinical sign for intracranial trauma (according to the Canadian CT Head Rule), or anticoagulation (right). The width of the bars represents the percentage of patients. The diagram was created using the software R [26]

According to the CCHR, only 26 cases (3.8%) fulfilled the criteria for CT for trauma evaluation, and 25 of these received a CT scan. In one patient with a unilateral periorbital haematoma, no head CT was performed; however, CT of the midface was ordered and ruled out a fracture. There were three patients with trauma and anticoagulation, all of whom received a CT. Figure 1 illustrates the decision for CT in (a) patients with or without a seizure-related fall or trauma and (b) in patients with seizure-related trauma and, either at least one clinical sign for intracranial trauma according to the CCHR, or anticoagulant therapy.

### Trauma consultation for other head injuries

Thirteen patients with visible head injuries were not referred to traumatology; in 81 cases (11.9%), seizure-related head injury led to traumatology consultation in the ED. Most of these were mild head injuries consisting of lacerations or contusions (n = 42/81, 51.9%). In one case (mentioned above) a skull base fracture was diagnosed. In another nine patients (11.1%), facial bone fractures were diagnosed involving the nasal bone in seven and the orbital floor in two cases. All facial bone fractures were treated conservatively in the ED, an otorhinolaryngologist recommended elective surgical treatment in two cases.

## Discussion

In our analysis of almost 700 patients with epileptic seizures seen in the ED over almost 2.5 years, a head CT was ordered in approximately half of all patients, although a CT indication for identifying trauma-related pathology—by CCHR or current anticoagulant medication in the context of seizure-related head impact—existed in less than 4%. None of the patients had critical traumatic brain injury; in two cases clinically less relevant findings were detected. In about 40% of patients with head impact, there were generally minor injuries in the form of cranial soft tissue contusions or lacerations.

Our results are in line with previously reported data: Welte et al. report cerebral CT imaging in nearly 70% of ED patients with seizures [35]. In their cohort, none of the patients with known epilepsy had a new acute pathology on imaging that needed immediate further treatment. Kwam and coauthors report acute findings in 8% of scans in ED visits for patients with epilepsy who underwent emergent neuroimaging [18]. In 2004, Lawn et al. analysed seizure-related injuries other than orolingual trauma occurring as a direct result of a seizure event [19]. During follow-up, 39 of the 247 persons with epilepsy (15.8%) had at least one seizure-related injury with mild head injuries constituting over two-thirds of cases. In a study published in 2002, Mower et al. enrolled 875 patients with new-onset seizure imaged in the ED and found emergent lesions in 81 cases (9.3%) [22]. In a prospective study with almost 28,000 seizures (predominantly with known epilepsy and 45% with a fall), 2.7% of patients were found to have a head injury (6.1% of those with a fall); one patient each had a skull base fracture, an epi- and a subdural haemorrhage [28].

We observed a high degree of variability and unpredictability related to physicians’ CT ordering practice with respect to circumstantial factors, clinical signs or symptoms and medical risk factors. Of all patients with some type of head impact, about two thirds did not receive a CT while one third did. Similarly, of all patients without risk factors for TBI (by CCHR or in a constellation of head impact and anticoagulation) a CT was obtained in approximately 50%. These findings merit discussion against the backdrop of existing recommendations and guidelines for CT use after head trauma, in particular the heterogeneity of TBI definitions and existing clinical tools as well as their applicability in and transferability to seizure patients. TBI is highly prevalent, still there is a notable terminological divergence and usage-related imprecision as, despite definitional differences, terms like TBI, concussion, mild or minor head trauma, and mild head injury are often used interchangeably. This in turn may lead to heterogeneous patient populations particularly in studies designed to determine the need for head CT in patients with suspected TBI [1]. The 2023 Clinical Policy of the American College of Emergency Physicians defines mild TBI as blunt head injury in patients aged 16 years or older with a GCS score of 14 or 15 and improvement to a GCS score of 15 at two hours postinjury (if the initial GCS score was 14), with or without a history of the following: loss of consciousness, amnesia, or disorientation presenting for evaluation within 24 h. It bears mentioning that factors other than trauma and subsequent TBI may cause alterations in mental state at the time of head injury and may thus pose a particular diagnostic challenge. This is especially true for seizures—even more so in that the diagnosis of TBI cannot be reconciled with a primary seizure event leading to blunt head injury because postictal patients may formally fulfil several TBI criteria. For these reasons, patients with a seizure preceding head trauma were explicitly excluded from investigations of clinical decision rules of CT utilisation for traumatic injury rule-out [23, 33].

As currently no decision tools exist for patients with seizures and the dedicated issue of seizure-related injury, we chose to apply the criteria of the CCHR to our subset of patients with minor head trauma to reappraise the need for CT imaging. The CCHR is recommended to provide decision support and improve head CT utilization in adults with a minor head injury [1]. We are aware that the application of a decision tool to a patient population excluded in the study deriving the tool itself may lead to both over-triage and unnecessary CT use, as well as under-triage and missed injuries. However, we only applied the CCHR to patients with head trauma and in conjunction with a dedicated neurological evaluation and interpretation of the clinical picture. This is particularly relevant with respect to potentially confounding clinical characteristics of the postictal state when patients may temporarily manifest neurological deficits and/or psychiatric symptoms [25]. In none of the cases in our investigation was the CCHR item “*Failure to reach GCS score of 15 within 2 h after injury*” present, as all cases with prolonged somnolence either recovered within a shorter time or were diagnosed as status epilepticus by EEG. Similarly, all observed neurological deficits in our sample resolved quickly and were evaluated as Todd’s paralysis.

Pragmatically, if patients show prolonged somnolence, they should be evaluated for the effects previously applied sedatives and closely monitored for neurological improvement. In addition, nonconvulsive status, which is far more frequent than TBI [31], must be considered as the primary differential diagnosis if patients fail to improve. In this situation, EEG should be performed. It is suitable for the verification of both a postictal state and a status epilepticus [2, 31] but currently used less often than emergent brain imaging. It must be acknowledged that emergent EEG may not be acquired in every ED, potentially necessitating further patient referral. In this situation, erring on the side of safety and obtaining a CT prior to transport may be a valid strategy. Finally, anticoagulant therapy is associated with a higher risk of intracranial haemorrhage after mild head trauma, so a head CT is recommended in these patients [15].

**Fig. 2:**
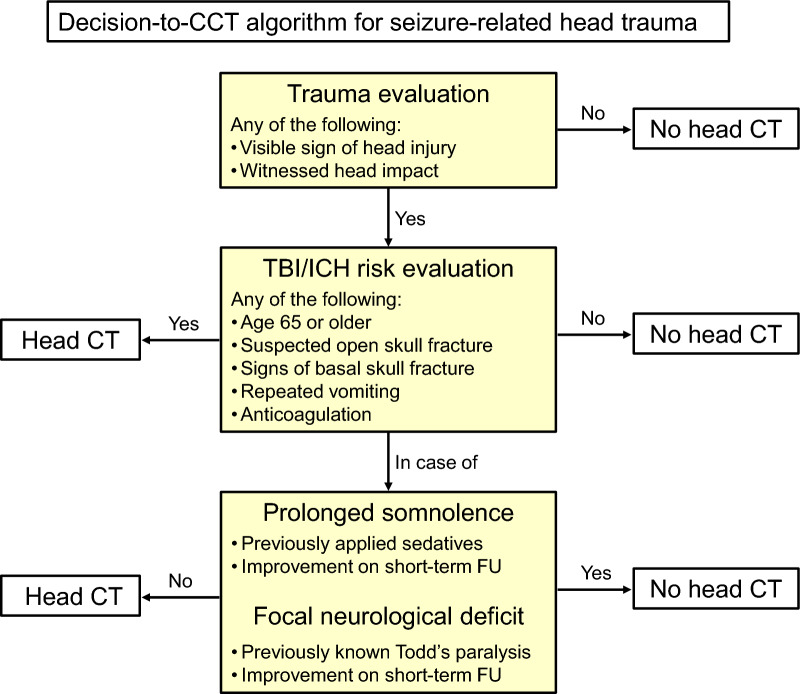
Decision-to-CT algorithm for seizure-related head trauma. This algorithm depicts the suggested approach to emergent head CT imaging in patients with seizure-related trauma. CCT, cranial computed tomography; FU, follow-up; ICH, intracranial haemorrhage; TBI, traumatic brain injury

In light of the results of our study and similar findings of others [18, 35] as well as the gaps and limitations of current clinical decision tools and recommendation with respect to the approach to head-injury in seizure-related trauma, we would like to suggest the following: if there was a trauma (i.e. any visible sign or reliable history of head impact), the CCHR for TBI risk evaluation should be applied and the previously mentioned clinical scenarios considered (see Fig. 2 for a proposed procedural algorithm). In light of the comparatively high frequency of seizure-associated injuries of the spine and axial skeleton [34, 36] traumatology consults should be initiated at a low-threshold [11]. It also requires mentioning that epileptic seizures with relevant trauma due to falls or head impact also occur frequently during hospital care and have a similarly low risk of intracranial injuries [10]. Hence, the suggested algorithm should also be applied in this scenario, where clinicians might be even less hesitant to order a CT.

Decision-making, particularly in the ED, is not only driven by medical factors. The specific circumstances and challenges of emergency medicine such as time- and resource-constraints, paucity of information and uncertainty intolerance appear to be equally impactful in this regard [24]. They may furthermore substantially contribute to reduced adherence to clinical decision rules, foster the “ruling out” of worst-case conditions—a frequently used approach to clinical decision-making in emergency physician training and practice—even in the absence of risk-mediating or predisposing factors, and thus result in overdiagnosis [17, 30, 32]. Strategies to manage these non-medical determinants of physician behaviour should therefore complement clinical decision tools in a meaningful way [21, 27].

Judicious use of CT examinations, which lead to a relatively high radiation exposure [4] in patients with epilepsy would positively impact the general risk–benefit relation particularly in light of the extremely low diagnostic yield in this patient population.

Our results must be interpreted in the context of several limitations. First, this study is a retrospective cohort study, and as such, subject to the characteristic limitations of using retrospective data, especially as it critically relies on the accuracy of patient records obtained from the hospital electronic database. Second, the study was conducted as a single-centre analysis; therefore, the results might not be generalizable to other EDs with different organizational structures. Finally, as cases were selected on a DRG-derived basis, patients with acute symptomatic seizures or patients with psychogenic non-epileptic seizures receiving a final coding not meeting our inclusion criteria would have been missed and not included in the study sample. This again may limit the generalisability of our results; however, we intend to provide auxiliary, not exclusive, recommendations for decision support in patients with seizures.

## Conclusion

The absence of clear recommendations regarding imaging for trauma apparently provokes frequent diagnostic rule-out even in patients with low risk for traumatic brain injury. We suggest an approach to identify patients not requiring a head CT by considering the CCHR, presence of anticoagulation and appreciating the postictal state as a feature specific to patients with seizures. We hope this proposal, aimed at complementing existing guidelines and recommendations for imaging in patients with seizures [6, 12], encourages further discussion and research on the rational and risk/cost–benefit-balanced utilisation of emergent imaging in seizure patients.

## Data Availability

Data are available from the corresponding author upon reasonable request.
